# What Increases Smokers’ Stress? Degree of Nicotine Dependence and Motivation to Quit Smoking in People After Myocardial Infarction

**DOI:** 10.3390/jcm14051545

**Published:** 2025-02-25

**Authors:** Robert Jan Łuczyk, Anna Ślifirczyk, Weronika Sieńska, Marta Łuczyk, Monika Baryła-Matejczuk, Kamil Sikora, Agnieszka Wawryniuk, Katarzyna Sawicka

**Affiliations:** 1 Department of Internal Medicine and Internal Nursing, Faculty of Health Sciences, Medical University of Lublin, 20-093 Lublin, Poland; luczykrobert@op.pl (R.J.Ł.);; 2 Faculty of Medical and Health Sciences, Institute of Health Sciences, University of Siedlce, 08-110 Siedlce, Poland; 3 Long-Term Care Nursing Department, Chair of Preventive Nursing, Faculty of Health Sciences, Medical University of Lublin, 20-093 Lublin, Poland; 4 Institute of Psychology and Human Sciences, WSEI University, 20-209 Lublin, Poland

**Keywords:** stress, smoking, myocardial infarction, motivation

## Abstract

**Background**: Myocardial infarction (MI) represents one of the major causes of hospitalization in cardiology departments, while tobacco smoking remains a significant public health challenge in Europe. Therefore, there is a pressing need to study this phenomenon in order to undertake preventive actions and thereby reduce the number of people smoking tobacco and, consequently reducing the rate of morbidity and disease. This study aims to assess the factors that increase stress and examine the strategies for coping with stress, nicotine addiction, and the motivation to quit smoking among patients after myocardial infarction. **Methods**: We analyzed 100 post-MI patients using a sociodemographic questionnaire, the Schneider Smoking Cessation Motivation Test, the Fagerstrom Test for Nicotine Dependence, and the Mini-COPE questionnaire. **Results**: A sense of helplessness emerged as the primary stress trigger (*p* = 0.012), with job loss, workplace issues, illness, family death, and relationship problems (*p* < 0.001) as key stressors. Post-MI patients showed a high degree of smoking cessation motivation, this was enhanced by limited cigarette access and social support. Patients primarily used self-distraction and behavioral disengagement (*p* < 0.001) as coping mechanisms. A higher degree of nicotine tolerance (rho = −0.355; *p* < 0.00) and increased stress frequency (rho = −0.169; *p* = 0.093) correlated negatively with cessation motivation. **Conclusions**: Post-MI patients demonstrate significant stress-related helplessness, particularly within the professional, family, and relationship domains. The prevalence of coping mechanisms centred around avoidance suggests the need for targeted psychological interventions in this population.

## 1. Introduction

### 1.1. Smoking and Coronary Artery Disease

Smoking remains one of the primary modifiable risk factors for cardiovascular disease, particularly myocardial infarction (MI). Despite well-documented health risks, a significant number of patients continue to smoke even after experiencing MI, which substantially increases their risk of recurrent cardiac events and mortality [1]. The relationship between smoking, stress, and cardiovascular disease creates a complex cycle that warrants detailed investigation, especially in post-MI patients.

Recent research has highlighted a phenomenon known as the “smoker’s paradox”, where smokers paradoxically show better short-term outcomes after acute MI compared to non-smokers. Paradossi et al. [2] demonstrated this effect in patients undergoing primary percutaneous coronary intervention, although it was emphasized that this apparent benefit is overshadowed by smoking’s overall detrimental effects on cardiovascular health. This paradox adds complexity to our understanding of smoking cessation motivation in post-MI patients and underscores the importance of investigating stress factors and cessation strategies in this population.

Smoking is considered to be one of the primary modifiable risk factors in the development of cardiovascular disease, particularly coronary artery disease [3]. Studies indicate that smokers face a 2–4 times higher risk of myocardial infarction compared to nonsmokers [4]. The mechanism of this phenomenon is complex and includes both the impact on the coagulation system—increased platelet activity [5,6], the increased fibrinogen level [7,8], and the intensification of the thrombotic processes [9,10]—as well as changes in the vascular system—damage to the vascular endothelium [9,11], the acceleration of the atherosclerotic process [12,13], and increased arterial stiffness [14].

### 1.2. Stress in the Context of Nicotine Addiction

The relationship between stress and smoking is bidirectional [15]. This is manifested in complex biological and psychological mechanisms. According to the biological basis of the effects of nicotine, it affects the hypothalamic–pituitary–adrenal axis and stimulates the secretion of corticoliberin (CRH) in the hypothalamus [1]. In addition, it increases the secretion of adrenocorticotropic hormone (ACTH) and increases the level of cortisol in the blood [11]. Therefore, there are disorders in the feedback mechanisms regulating the level of stress hormones [16]. The dopaminergic system is modulated. This means that there is a direct activation of dopaminergic neurons in the mesolimbic system, increased dopamine release in the nucleus accumbens, and changes in the expression of dopamine receptors [10,12,17]. This is accompanied by the development of reward system sensitization [16]. In turn, changes in the activity of the sympathetic nervous system include the increased secretion of adrenaline and noradrenaline, an increased heart rate and blood pressure [18], disturbances in the regulation of the vascular tone, and the impact on glucose and lipid metabolism [19].

The psychological mechanisms of understanding the relationship between stress and nicotine addiction indicate that smoking is identified as a way of coping with stress. The process involves, among other factors, the development of a conditioned (learned) response to stressful situations [20] and the subjective feeling of tension reduction after smoking a cigarette [21]. In the case of nicotine addiction, we must account for the development of behavioral automatisms in stressful situations [22]. Numerous studies also show that during periods of abstinence, smokers experience a significant increase in stress levels. This leads to increased anxiety and depressive symptoms [4], increased irritability, and concentration problems [1], as well as sleep and appetite disorders [23]. Addicts experience difficulties in regulating emotions [4,24]. A so-called vicious circle is observed: stress → smoking → relief → greater susceptibility to stress [25]. In addition, the mechanisms of avoiding negative emotional states are reinforced and natural mechanisms of coping with stress are weakened [26].

### 1.3. Motivation to Quit Smoking

The literature on the subject indicates several key aspects of the motivation to quit smoking. Factors that enhance motivation include an awareness of health risks, social support, positive experiences from previous attempts to quit smoking, as well as critical events (e.g., heart attack) [27]. The causes of the various difficulties in quitting smoking are indicated as high levels of physical dependence, fear of withdrawal symptoms, life stress, or a lack of confidence in one’s own abilities [28,29]. Patients after a heart attack constitute a special group in the context of nicotine addiction [30,31,32] due to their acute sense of their life being threatened, increased levels of anxiety, or depressive symptoms. Therefore, effective support for patients after a heart attack requires a comprehensive approach [1]. The intersection of smoking dependency, stress, and motivation to quit becomes particularly relevant in the post-MI population. Research has demonstrated that patients who experience MI often initially show a high degree of motivation to quit smoking, but this motivation frequently decreases over time, coinciding with increased stress levels [16,28,29]. Understanding the factors that influence this dynamic is crucial for developing effective interventions. Previous studies have primarily focused on either smoking cessation programs or stress management in post-MI patients separately. However, there is limited research available which examines how nicotine dependence levels and motivation to quit smoking interact to influence stress levels in this specific population. This knowledge gap is significant, as understanding these relationships could inform more effective, targeted interventions for this high-risk group. Therefore, this study aims to investigate the relationship between nicotine tolerance levels, the motivation to quit smoking, and the stress intensity experienced by post-MI patients.

## 2. Materials and Methods

### 2.1. Study Design and Population

This study was conducted among patients treated at the Military Clinical Hospital in Lublin in the Cardiology Department of the Cardiac Intensive Care Unit and in the Hemodynamics Laboratory. The study group consisted of patients who were smokers and had suffered a myocardial infarction. Of the 107 initially screened patients, 100 were included in the final analysis. Seven patients were excluded due to incomplete questionnaire responses (n = 5) and withdrawal of consent (n = 2). The consent to conduct this study was obtained from the Hospital Commander and the participants (patients). Participants were recruited during their initial hospitalization following MI (within 6–8 days of the acute event). This timing may influence the interpretation of stress levels and the cessation of motivation, as both factors could be temporarily elevated due to the recent acute cardiac event.

The following research tools were used in this study: an original questionnaire—a personal data sheet—Test of Motivation for Ceasing Smoking by Nina Schneider, Fagerstrom Test For Nicotine Dependence, and the Mini-COPE questionnaire.

An original questionnaire—contains information on the socio-demographic status of the participants and basic questions about their disease.

Test of Motivation for Ceasing Smoking by Nina Schneider [33]—12 questions with YES or NO answers. It concerns the respondents’ motivation to stop smoking. The predominance of YES answers indicates a high level of motivation to plan to stop smoking cigarettes.

Fagerstrom Test For Nicotine Dependence (FTND) [34]—a test that allows one to assess with a high degree of probability whether a given person is addicted to nicotine. The test consists of 6 questions. Each question has a value from 0 to 1 or from 0 to 2. The maximum number of points for the test is 11. Obtaining 0–4 points means the absence of nicotine addiction features, 5–8 points indicate the presence of nicotine addiction, while 9–11 indicate unequivocal symptoms of pharmacological nicotine addiction and some diseases which are related to smoking.

Mini-COPE [35,36]—a tool used to measure stress-coping strategies. It consists of 28 statements regarding the typical behaviour of people in difficult situations. The respondent answers on a 4-point scale: 1 = I almost never do this, 2 = I rarely do this, 3 = I often do this, and 4 = I almost always do this.

### 2.2. Statistical Analysis

All statistical analyses were performed using SPSS Statistics 29 software with a significance level of α < 0.05. The statistical analysis framework comprised several complementary approaches. For descriptive statistics, we calculated the values of a central tendency and dispersion (mean, standard deviation, and median) along with distribution characteristics (skewness and kurtosis coefficients). Data visualization was accomplished through bar graphs, scatterplots, and histograms. The distribution analysis included a normality assessment using Kolmogorov–Smirnov tests, supported by a visual inspection of histograms and an evaluation of skewness and kurtosis coefficients.

For inferential statistics, we employed 95% confidence intervals to analyze the means of key variables including the motivation to quit smoking, nicotine addiction, and stress levels. Student’s *t*-tests were utilized for analyzing coping strategies, while binomial tests were applied to analyze the proportions of stress-related responses, using a reference probability of 0.5. In order to examine the relationships between the motivation to quit smoking, the level of nicotine addiction, the number of stressful situations, and coping strategies, we employed Spearman’s rho correlation coefficient. The relationships between smoking-related variables (motivation and nicotine addiction) and sociodemographic and medical characteristics were assessed using Kruskal–Wallis H tests.

This comprehensive statistical framework was applied to all questionnaire data obtained from the Schneider, Fagerstrom, and Mini-COPE instruments, as well as stress situation frequencies, thereby ensuring a robust and reliable analysis of the results.

## 3. Results

### 3.1. Characteristics of the Study Group

This study analyzed data from 100 respondents, with 52% women and 48% men. The age distribution of the participants was 30–40 years (19%), 41–50 years (17%), 51–60 years (20%), 61–70 years (25%), 71–80 years (15%), and over 80 years (4%). With regard to their place of residence, 36% lived in rural areas, while urban residents were distributed across cities of varying sizes: up to 30,000 inhabitants (21%), 30,000–100,000 inhabitants (24%), and over 100,000 inhabitants (19%).

The educational background of the participants comprised vocational (32%), secondary (29%), higher education (26%), and primary education (13%). Their professional status was distributed among white-collar workers (32%), manual workers (22%), retirees (21%), disability pensioners (16%), and unemployed individuals (9%). Their marital status showed that 56% were married, 22% widowed, 13% single, and 9% divorced.

Regarding their cardiac history, 60% of participants had experienced one myocardial infarction, 30% two episodes, and 10% three or more episodes. The time elapsed since their most recent infarction was one month (16%), three months (21%), six months (23%), one year (21%), and more than one year (19%). The smoking duration among the participants ranged from less than 10 years (15%), 10–20 years (33%), 21–30 years (21%), 31–40 years (17%), to over 40 years (14%).

### 3.2. Characteristics of Situations That Increase Stress and Strategies for Coping with It

The subject of the following subsection is the characteristics of the stress experienced by the respondents as well as the sources and intensifying factors of that stress.

Table 1 presents the main causes of stress experienced by the respondents. In the column of frequencies and percentages, the number of people who experienced a given cause of stress is presented.

A statistically significant minority of respondents experienced stress related to job loss, problems at work, the death of a family member, the illness of a family member, and divorce and relationship problems; none of the respondents mentioned other sources of stress. It was not shown that a statistically significant minority or majority of respondents experienced stress related to the deterioration of their own health.

Table 2 presents the situations experienced by the respondents that, in their opinion, intensified their stress.

A statistically significant minority of respondents mentioned the lack of help from the environment as a stress increasing situation. A statistically significant majority of respondents assessed that the feeling of helplessness increased their sense of stress. Among other situations that increased the sense of stress, the respondents mentioned a lack of agreement (2%), quarrels (1%), loneliness (7%).

The average number of stressful and stress-intensifying situations was 3.24 [95% CI (2.99, 3.49)] with a standard deviation of 1.27. Half of the respondents experienced three or more negative situations. The Kolmogorov–Smirnov test showed statistically significant differences between the distribution of the number of stressful and stress-intensifying situations and the normal distribution (KS = 0.185; *p* < 0.001). The skewness and kurtosis coefficients (SKEW = 0.677; KURT = 0.956) did not indicate that these differences were very large, although a fairly high kurtosis was noted in the distribution.

Table 3 presents the stress-coping strategies used by the respondents in comparison to the strategies used by the COPE-normative group (the mean results of this group are presented in the “tested value” column).

The analyses conducted showed that the participants significantly more often coped with stress by using self-distraction and through behavioral disengagement and moderately more often by self-blame or turning to religion and denial. To a small extent (although still statistically significant), the participants more often turned to substance use, and less often reacted with humor, and also more often through acceptance or the use of instrumental support. No statistically significant differences were found between the participant and the COPE-normative group in the remaining strategies of coping with stress.

### 3.3. Degree of Nicotine Addiction and Motivation to Quit Smoking

The results obtained in the Schneider and Fagerstrom tests are presented below. They indicate the degree of nicotine addiction and motivation to quit smoking. In addition, Table 4 presents the factors that helped the patients to quit smoking.

The factor that helped respondents to stop smoking for almost half of their number (47%) was being around non-smokers, another 37% of the respondents reported that they were helped by an increased workload, 26% by setting life goals, 15% by practicing sports, and 8% by being alone. More than half of the respondents (59%) had difficulty using cigarettes due to their limited access to them, and more than half (53%) through support from loved ones. Among other factors, the respondents mentioned the high price of cigarettes (3%) and spending time with grandchildren (1%).

The average result on the nicotine tolerance scale was 5.45 [95% CI (4.96, 5.94)] with a standard deviation of 2.46. Half of the respondents obtained results no higher than 5. The Kolmogorov–Smirnov test showed statistically significant differences between the distribution on the nicotine tolerance scale and the normal distribution, KS = 0.132; *p* < 0.001, while the skewness and kurtosis coefficients (SKEW = 0.288; KURT = −0.48) did not indicate that these differences were very large. The population of respondents had a moderate level of nicotine addiction. A low level of nicotine addiction was a characteristic for 41% of respondents, moderate for 49% of respondents, and high for 10% of respondents.

The average score on the motivation to stop smoking scale was 1.78 [95% CI (0.79, 2.77)] with a standard deviation of 5.01. Half of the respondents obtained scores higher than or equal to 2. The Kolmogorov–Smirnov test showed statistically significant differences between the distribution on the motivation to stop smoking scale and the normal distribution (KS = 0.095; *p* = 0.026). The skewness and kurtosis coefficients (SKEW = −0.289; KURT = −0.024) also, in this case, do not indicate that these deviations were significant.

Negative results mean a lower motivation to quit than the motivation to continue this addiction. Positive results mean a predominance of the motivation to quit smoking. The 95% confidence interval indicates that in the studied population, there is a predominance of motivation to stop smoking [95% CI (0.79, 2.77)]. The median indicated that half of the respondents obtained results higher than or equal to two.

The correlation coefficient indicates a moderate, negative relationship between nicotine tolerance and the motivation to stop smoking (rho = −0.355; *p* < 0.001). The higher the nicotine tolerance, the lower the motivation to stop smoking.

### 3.4. Stress and Motivation to Stop Smoking and Level of Nicotine Addiction

The analyses conducted did not show any statistically significant correlations between the number of stressful situations and nicotine tolerance (rho = −0.012; *p* = 0.904) and the motivation to stop smoking (rho = −0.169; *p* = 0.093). It is worth noting that in the case of correlations between the motivation to stop smoking and the number of stressful situations and situations that increase the level of stress, the result of the correlation coefficient was close to the statistical significance, which could indicate that the higher the number of stressful situations, the lower the motivation to stop smoking.

The analyses conducted revealed statistically significant correlations between nicotine tolerance and active coping (weak correlation), planning (weak correlation), the use of instrumental support (weak correlation), the use of emotional support (moderate correlation), turning to religion (moderate correlation), self-distraction (weak correlation), behavioral disengagement (weak correlation), and substance use (moderate correlation) (Table 5).

The analyses conducted revealed statistically significant correlations between the motivation to stop smoking and active coping (moderate correlation), planning (weak correlation), the use of emotional support (weak correlation), substance use (weak correlation), self-distraction (weak correlation), and behavioral disengagement (moderate correlation). The higher the level of the motivation to quit smoking variable, the higher the level of active coping, planning, the use of emotional support, and self-distraction, and the lower the level of behavioral disengagement or substance use.

The analysis showed a statistically significant, weak relationship between the overload of responsibilities and the score obtained by the respondents on the nicotine tolerance scale (Table 6). The respondents who admitted that the overload of responsibilities was difficult for them were also characterized by a slightly lower level of nicotine tolerance. No statistically significant relationships were shown between nicotine tolerance and the remaining variables.

## 4. Discussion

Tobacco smoking is one of the biggest public health problems. The addiction develops on a biological, psychosociological, and clinical basis, and also brings about many negative consequences, including lung and larynx cancers, heart attacks, asthma, and chronic obstructive pulmonary disease. Many smokers try to quit smoking by adopting a state of readiness to take some specific action [37,38].

The aim of this study was to assess the factors that increase stress, strategies for coping with stress, nicotine addiction, and the motivation to quit among patients after myocardial infarction.

The analysis of the data collected during this study allows for the formulation of several key conclusions. Firstly, it was shown that the intensity of stress in patients after myocardial infarction is particularly high in situations when they feel helpless. This is important information for medical personnel to pay special attention to regarding the sense of being in control, agency, and self-efficacy in the process of rehabilitation and support for patients after a heart attack, which can significantly affect the level of stress experienced [39].

At the same time, studies have shown that after a heart attack patients who are active smokers are highly motivated to stop smoking. This observation is consistent with the results of previous studies, which indicates that the experience of a serious health incident, such as a heart attack, is a strong incentive to modify unfavorable health habits [37,40,41]. From a practical point of view, this indicates the need for effective support for these patients in quitting their tobacco addiction as a part of a comprehensive rehabilitation.

Another important conclusion is the observation that the most common sources of stress for patients after a heart attack are situations related to professional work and difficulties in interpersonal relations. This suggests that when working with a group of such patients, special attention should be paid to the issues of social and professional functioning, which constitute a significant challenge in the process of returning to health and full fitness [24,42].

With reference to the strategies of coping with stress used by the examined patients, it was shown that they most often use cognitive and behavioral mechanisms to distract attention and stop certain activities. This is an important clue for medical personnel while selecting appropriate forms of support and psychoeducation aimed at equipping patients with more adaptive ways of coping with difficult emotions and situations [24,43].

It is also worth noting that there is a relationship between nicotine tolerance and the motivation to stop smoking—the higher the tolerance, the lower the motivation. This observation is in line with the findings of other researchers who emphasize the importance of physiological addiction in the process of quitting smoking [44]. In turn, the statement that the more stressful the situations that the patients experience, the lower their motivation to quit smoking leads to the conclusion that a holistic approach to the problem is required, taking into account not only purely addiction issues, but also the psychosocial determinants of the health behaviors of this group of patients [45,46]. It is also worth mentioning that the timing of our assessment, during the acute post-MI period, may have influenced our findings regarding stress levels and cessation motivation. Future studies should consider a longitudinal assessment to differentiate between acute event-related and chronic stressors.

In summary, the conducted studies provide valuable information concerning the psychological functioning of patients after myocardial infarction, with particular emphasis on the issues of stress and coping with it, as well as the motivation to change unfavorable health habits. The obtained results may constitute an important framework for medical personnel involved in the comprehensive rehabilitation of this group of patients.

## 5. Conclusions

The findings from this study reveal several important patterns regarding stress, smoking behaviours, and coping mechanisms in post-myocardial infarction patients. Our analysis demonstrates that feelings of helplessness serve as the primary trigger for increased stress in this population, particularly in work-related contexts and interpersonal relationships. While these patients generally exhibit a high motivation to quit smoking, several factors influence their cessation efforts. Environmental factors play a crucial role, with difficultly in accessing cigarettes, support from family members, and exposure to non-smoking social circles facilitating cessation attempts.

In terms of stress management, patients predominantly employ avoidance-based coping strategies, primarily relying on self-distraction and behavioral disengagement. To a lesser extent, they engage in self-blame, religious practices, and denial as secondary coping mechanisms. Less frequently observed strategies include substance use, humor, acceptance, and seeking instrumental support. Notably, our findings indicate significant relationships between stress, nicotine dependence, and cessation motivation. Higher levels of nicotine tolerance correlate with a decreased motivation to quit smoking, while an increased frequency of stressful situations similarly diminishes cessation motivation.

These findings underscore the complex interplay between psychological stress, nicotine dependence, and smoking cessation efforts in post-MI patients, suggesting the need for comprehensive intervention strategies that address both stress management and nicotine dependence simultaneously. These preliminary findings are based on a sample of 100 patients. While these observations provide valuable insights, larger-scale studies are required to confirm these associations and their clinical significance.

## 6. Limitations

Several limitations of this study should be considered when interpreting the results. Firstly, the nature of the data collection prevents us from drawing conclusions about causal relationships between stress levels, smoking behaviors, and coping mechanisms over time. In addition, the reliance on self-reported measures may introduce a recall bias, particularly with regard to stress levels and smoking behaviours. The sample size of 100 participants, while adequate for a preliminary analysis, may limit the generalizability of our findings to the broader population of post-MI patients. Furthermore, our study did not account for potentially confounding variables such as pre-existing mental health conditions or concurrent medical treatments that might influence both stress levels and smoking cessation motivation. The single-centre design of this study may also limit the external validity of our findings, as treatment protocols and patient populations may vary across different healthcare facilities. Lastly, the assessment of coping strategies was conducted at a single time point, which may not capture the dynamic nature of coping mechanisms that patients employ throughout their recovery process. A significant limitation of our study is also the lack of follow-up data on actual smoking cessation success rates among participants. Future research should incorporate the longitudinal tracking of cessation outcomes to better understand how the measured variables (stress, nicotine tolerance, and motivation) predict successful quitting. Future longitudinal studies with larger, multi-centre samples would be valuable in addressing these limitations and providing more robust evidence for the relationships observed in our study.

## Figures and Tables

**Table 1:** Main cause of stress.

Main Cause of Stress	Frequency	Percent	*p*
Job loss	9	9	0.000
Problems at work	31	31	0.000
Death of a family member	23	23	0.000
Illness of a family member	24	24	0.000
Deterioration of one’s own health	44	44	0.271
Divorce	9	9	0.000
Relationship problems (quarrels, slander)	26	26	0.000
Other	0	0	0.000

*p*—test probability of the binomial test.

**Table 2:** Situations that increase stress.

Situations That Increase Stress	Frequency	Percent	*p*
Overload of responsibilities	41	41	0.089
Lack of help from the environment	29	29	0.000
Lack of support	51	51	0.920
Helplessness	63	63	0.012
Other	10	10	0.000

*p*—test probability of the binomial test.

**Table 3:** Stress coping strategies of the respondents.

Response to Stress	Tested Value	M	SD	Student’s *t*-Test			
				T	df	*p*	D
Active coping	1.87	1.88	0.69	0.146	99	0.884	0.015
Planning	1.89	1.94	0.66	0.680	99	0.498	0.068
Use of instrumental support	1.67	1.85	0.70	2.565	99	0.012	0.257
Use of emotional support	1.78	1.85	0.71	0.992	99	0.323	0.099
Self-blame	0.82	1.28	0.61	7.474	99	0.000	0.747
Religion	0.85	1.45	0.89	6.706	99	0.00	0.671
Positive reframing	1.66	1.61	0.66	−0.828	99	0.409	0.083
Venting	1.56	1.66	0.57	1.662	99	0.100	0.166
Acceptance	1.34	1.66	0.64	4.934	99	0.000	0.493
Denial	0.63	1.08	0.74	6.043	99	0.000	0.604
Self-distraction	1.01	1.67	0.62	10.568	99	0.000	1.057
Behavioral disengagement	0.37	1.06	0.64	10.717	99	0.000	1.072
Substance use	0.58	0.92	0.78	4.354	99	0.000	0.435
Humor	1.20	0.86	071	−4.872	99	0.000	0.487

M—mean; SD—standard deviation; *p*—test probability; T—Student’s *t*-test result; df—degrees of freedom; D—Cohen’s d effect size.

**Table 4:** Factors contributing to smoking cessation.

Factors Contributing to Smoking Cessation	Frequency	Percentage
Being around non-smokers	47	47
Increased workload	37	37
Practicing sports	15	15
Setting life goals	26	26
Being alone	8	8
Difficultly in accessing cigarettes	59	59
Support from loved ones	53	53
Other	5	5

**Table 5:** Coping with stress, nicotine tolerance, and the motivation to cease smoking.

Response to Stress	Nicotine Tolerance		Motivation to Quit Smoking	
	Rho	*p*	Rho	*p*
Active coping	−0.257	0.010	0.349	0.000
Planning	−0.236	0.018	0.248	0.130
Use of instrumental support	−0.238	0.017	0.110	0.276
Use of emotional support	−0.349	0.000	0.296	0.003
Self-blame	0.173	0.085	−0.021	0.835
Religion	−0.351	0.000	0.151	0.132
Positive reframing	−0.119	0.238	0.004	0.967
Venting	0.099	0.326	0.012	0.909
Acceptance	−0.067	0.509	0.127	0.210
Denial	0.129	0.202	−0.128	0.205
Self-distraction	−0.233	0.019	0.233	0.020
Behavioral disengagement	0.244	0.014	−0.356	0.000
Substance use	0.394	0.000	−0.213	0.034
Humor	0.125	0.216	0.111	0.273

Rho—Spearman’s rho coefficient; *p*—test probability.

**Table 6:** Nicotine tolerance and the causes of stress.

Variable Category	Variable	Yes/No	Nicotine Tolerance				Kruskal–Wallis H Tests			
			M	SD	N	Mr	H	df	*p*	ε^2^
Main cause of stress	Job loss	No	5.29	2.32	91	49.04	2.587	1	0.108	0.027
		Yes	7.11	3.30	9	65.22				
	Problems at work	No	5.62	2.49	69	52.17	0.746	1	0.388	0.008
		Yes	5.06	2.39	31	46.79				
	Death of a family member	No	5.35	2.50	77	48.76	1.224	1	0.269	0.013
		Yes	5.78	2.35	23	56.33				
	Illness of a family member	No	5.45	2.52	76	50.30	0.016	1	0.900	0.000
		Yes	5.46	2.30	24	51.15				
	Deterioration of one’s own health	No	5.73	2.34	56	53.88	1.750	1	0.186	0.018
		Yes	5.09	2.59	44	46.20				
	Divorce	No	5.38	2.42	91	49.68	0.829	1	0.363	0.009
		Yes	6.11	2.93	9	58.83				
	Relationship problems (quarrels, slander)	No	5.38	2.37	26	51.81	0.073	1	0.788	0.001
		Yes	5.65	2.73	100	0.00				
Stress-increasing factors	Overload of responsibilities	No	5.90	2.49	59	55.64	4.581	1	0.032	0.047
		Yes	4.80	2.29	41	43.11				
	Lack of help from the environment	No	5.34	2.37	71	49.12	0.563	1	0.453	0.006
		Yes	5.72	2.68	29	53.88				
	Lack of support	No	5.10	2.13	49	47.32	1.175	1	0.278	0.012
		Yes	5.78	2.72	51	53.56				
	Helplessness	No	5.41	2.37	37	49.73	0.042	1	0.838	0.000
		Yes	5.48	2.53	63	50.95				
	Other	No	5.46	2.43	90	50.30	0.043	1	0.835	0.000
		Yes	5.40	2.84	10	52.30				

N—number of observations; M—Mean; SD—standard deviation; Mr—mean rank; H—test result; *p*—test probability; df—degrees of freedom; ε^2^—epsilon squared.

## Data Availability

The availability of the data should be personally requested from the corresponding author at monika.baryla@wsei.lublin.pl.
